# Role of long non-coding RNA-adducin 3 antisense RNA1 in liver fibrosis of biliary atresia

**DOI:** 10.1080/21655979.2022.2041321

**Published:** 2022-03-04

**Authors:** Yongqin Ye, Weifang Wu, Jiachen Zheng, Lihui Zhang, Bin Wang

**Affiliations:** a Department of General Surgery, Shenzhen Children’s Hospital, Shenzhen, China; b Department of Pediatric Surgery, Shantou University Medical College, Shantou, China; c Department of Traditional Chinese Medicine, Shenzhen Children’s Hospital, Shenzhen, China

**Keywords:** Long non-coding RNA, lnc-ADD3-AS1, LX-2 cell proliferation, LX-2 cell migration, liver fibrosis, biliary atresia

## Abstract

Biliary atresia (BA) is a devastating liver disease in neonates. Liver fibrosis is regarded as a universal and prominent feature of BA. Studies have revealed that long non-coding RNAs (lncRNAs) regulate cellular processes during the development of liver fibrosis in BA. Long non-coding RNA-adducin 3 antisense RNA1 (lnc-ADD3-AS1) has been shown to increase susceptibility to BA. However, the role of lnc-ADD3-AS1 in liver fibrosis in BA remains unclear. Here, we investigated the role of lnc-ADD3-AS1 in the proliferation, migration, and apoptosis of the immortalized human hepatic stellate cell (HSC) line, LX-2. We successfully overexpressed and silenced lnc-ADD3-AS1 in LX-2 cells using adenovirus vectors and evaluated the proliferation of transfected cells using the Cell Counting Kit-8 (CCK8) assay. Cell apoptosis was detected using annexin V–fluorescein isothiocyanate (FITC)/propidium iodide (PI) double staining and flow cytometry. We then analyzed cell migration by performing wound-scratch and transwell migration assays. Our results show that lnc-ADD3-AS1 significantly promoted LX-2 cell proliferation and attenuated apoptosis. More importantly, lncRNA-ADD3-AS1 significantly accelerated the migration of LX-2 cells. Our data indicated that lncRNA-ADD3-AS1 plays a role in the pathogenesis of liver fibrosis in patients with BA and may serve as a potential diagnostic marker for monitoring liver fibrosis in BA or as a therapeutic target for the disease.

## graphical abstract

**Figure:**
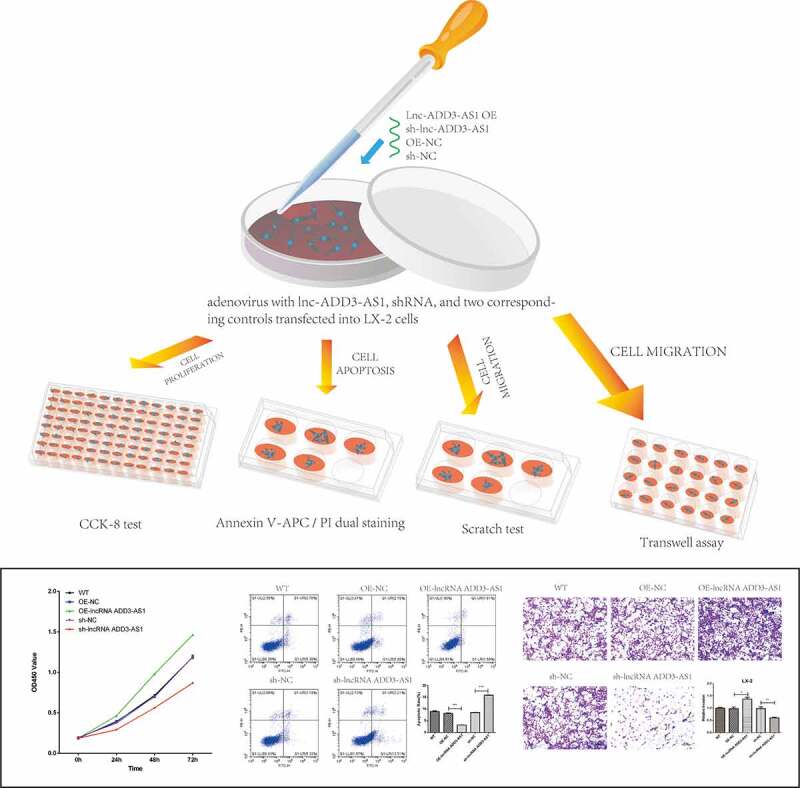

## Introduction

Biliary atresia (BA) is a severe liver disease in neonates, and it has a high incidence rate in China. It usually leads to liver failure and even death within a few years of birth due to fibrosclerosis and obliteration of bile ducts if left untreated [1–6]. Liver fibrosis is not only regarded as a universal and prominent feature of BA but also the most important predictor of outcome following portoenterostomy [7,8]. Although Kasai hepatoportoenterostomy (KHPE) helps prevent the development of cirrhosis and improves long-term survival, there is still an urgent requirement to identify the mechanisms underlying fibrogenesis and reliable therapeutic targets for liver fibrosis in BA.

Increasing evidence has revealed that non-coding RNAs, including microRNAs and long non-coding RNAs (lncRNAs), play key roles in regulating liver-related fibrosis and disease [9,10]. Indeed, microRNAs such as microRNA-21 have been reported to affect liver fibrosis in patients with BA [11]. LncRNAs are RNA transcripts longer than 200 nucleotides without protein-coding ability [12]. The lncRNA Alu-mediated p21 transcriptional regulator (APTR) was shown to promote hepatic stellate cell (HSC) activation and the progression of liver fibrosis [13]. Aberrant APTR expression has also been observed in the liver of BA affected infants, indicating that APTR may contribute to liver fibrogenesis [14]. It has been shown that lincRNA-p21 (hepatocyte long intervening non-coding RNA-p21) promotes p21 expression and inhibits the cell cycle proliferation and progression of primary HSCs [15]. Other studies have demonstrated that lincRNA-p21 is significantly upregulated during liver fibrosis [16] and was initially considered to be a transcriptional target of p53 [17]. Protein TET3 (ten-eleven translocation 3) was closely associated with HSC activation, and lncRNA HIF1A-AS1 expression was significantly increased in TET3 knockdown LX-2 cells [18]. Further studies have shown that silencing of lncRNA HIF1A-AS1 reduces apoptosis and promotes LX-2 cell proliferation. These results illustrate the role of TET3 in HSC activation by modulating HIF1A-AS1 expression [18]. Surprisingly, 34,146 differentially expressed (DE) lncRNAs were identified during human HSC quiescence and activation, and the expression of only 1685 DE mRNAs and 3763 DE lncRNAs changed significantly after activation [19]. Therefore, we need to identify and characterize more lncRNAs that participate in the progression of liver fibrosis in BA.

Membrane skeletal protein adducin 3 (ADD3) is abundantly expressed in the biliary tract of the fetal liver and was shown as a susceptibility gene of BA in both Asian and Caucasian populations by a genome-wide association study (GWAS) [20–22]. By knocking down ADD3 in zebrafish, Tang *et al*. identified ADD3 as a putative genetic risk factor for BA susceptibility that may affect the Hedgehog pathway, which is an important factor in BA pathogenesis [23]. In our previous study, we found that miR-145 contributes to liver fibrosis in BA by upregulating ADD3 expression [24]. Interestingly, a long non-coding RNA ADD3-antisense 1 (lncRNA-ADD3-AS1), situated in locus 10q25.1, with the 5’-end overlapping with the ADD3 gene, was shown to increase susceptibility to BA, which suggested that the lncRNA-ADD3-AS1 transcript played an additive role in the pathogenesis of the disease [25]. Nevertheless, the functions of ADD3 and lnc-ADD3-AS1 in liver fibrosis during BA are unclear, and the underlying mechanisms need to be elucidated.

In the present study, we aimed to determine the role of lncRNA-ADD3-AS1 in the proliferation, migration, and apoptosis of immortalized human HSCs and LX-2 cells. We successfully overexpressed and silenced lnc-ADD3-AS1 in LX-2 cells using adenovirus vectors and evaluated the proliferation of transfected cells using the Cell Counting Kit-8 (CCK8) assay. Apoptosis was detected using annexin V–fluorescein isothiocyanate (FITC)/propidium iodide (PI) double staining and flow cytometry. We then analyzed cell migration by performing wound-scratch and transwell migration assays. Our results showed that lnc-ADD3-AS1 significantly promoted LX-2 cell proliferation and attenuated apoptosis. Importantly, lncRNA-ADD3-AS1 significantly accelerated the migration of LX-2 cells. Moreover, our gain- and loss-of-function studies demonstrated that lncRNA-ADD3-AS1 played a regulatory role in the progression of liver fibrosis in BA. Therefore, LncRNA-ADD3-AS1 could be considered as a potential therapeutic target for this disease.

## Materials and methods

### Cell culture and transfection

LX-2 cells were cultured in Dulbecco’s modified Eagle’s medium (DMEM, Corning, USA) containing 10% fetal bovine serum (FBS), penicillin G (100 U/mL), and streptomycin (100 μg/mL) at 37°C with 5% CO_2_ in a humidified atmosphere.

Cells were seeded the day before transfection and grown to 50–70% confluence. Lnc-ADD3-AS1 overexpressing (OE-ADD3-AS1), short hairpin RNAs (shRNAs) specifically targeting lnc-ADD3-AS1 (sh-ADD3-AS1), or the corresponding controls (OE-NC or sh-NC) were delivered into LX-2 cells using an adenovirus vector. Untreated wild-type (WT) cells were used as negative controls. For lnc-ADD3-AS1 overexpression, primers used were as follows:

Forward primer: AGGTCGACTCTAGAGactcgagcggccgcagtgc;

Reverse primer: TGTAGTCCATACCGGtaaacttaagcttttcagtggcagag

For lnc-ADD3-AS1 silencing, specific shRNAs were used as follows.

Lnc-ADD3-AS1-1, gagacaaggaaattgaaatttcaagagaatttcaatttccttgtctctttttt;

Lnc-ADD3-AS1-2, gatgttccctatagtaaatttcaagagaatttactatagggaacatctttttt;

Lnc-ADD3-AS1-3, ggcctcatttgaaccacatttcaagagaatgtggttcaaatgaggcctttttt.

### Cell proliferation assay

The assay was performed as previously described [26]. Briefly, transfected LX-2 cells were seeded into 96-well plates at a density of 1000 cells per well and cultured continued for 0, 24, 48, and 72 h respectively. Subsequently, 10 μL CCK-8 solution (Solarbio) was added to each well and incubated at 37°C for 30 min. The optical density at 450 nm, indicative of a positive correlation with cell viability, was measured using a microplate reader (MB-580; Heales).

### Flow cytometry analysis

The assay was performed as previously described [26]. Briefly, LX-2 cells were cultured and transfected with the indicated adenovirus vector for 24 h, after which the cells were digested with EDTA-trypsin and washed with PBS. Annexin V-FITC/PI dual staining (Sigma, USA) was performed to determine LX-2 cell apoptosis, following the manufacturer’s instructions. Apoptotic cells were measured using a FACS flow cytometer (Beckman Coulter, CytoFLEX S) and analyzed using the Flow Jo software (Becton Dickinson, CA, USA).

### Scratch test

The assay was performed as previously described [26]. Briefly, the transfected LX-2 cells were grown to 100% confluence in 6-well plates and then scraped using a 100 μL pipette tip. The wells were washed three times with PBS, and fresh medium was added. The plate was incubated at 37°C and the cells were allowed to migrate into the wound and then fixed. The migration distance of the cell-scratched region was examined under an inverted microscope after 0, 24, and 48 h. The migration ratio (%) was calculated as the width at 24 h and 48 h divided by the width at 0 h.

### Transwell assay

Cell migration was evaluated in 24-well transwell chambers (Corning, MA, USA) according to the manufacturer’s instructions. Briefly, the lower chambers of a 24-well plate were filled with 500 μL of DMEM containing 10% FBS. Transfected cells (1 × 10^4^) in 100 μL of medium without FBS were placed in the upper compartment of the wells. The transwell chambers were incubated at 37°C in 5% CO_2_ humidified atmosphere for 24 h. The cells that invaded the lower surface of the polycarbonate membranes (12 μm pore size) were fixed with 4% paraformaldehyde for 15 min and stained with 0.5% crystal violet solution for 20 min. The number of cells was measured under an inverted microscope and quantified by counting three microscopic fields/filter.

### Statistical analysis

GraphPad Prism 6.0 was used to evaluate statistical analysis. All results were represented as mean ± standard error of the mean (SEM) from three individual tests. Statistical comparisons were evaluated using unpaired Student’s t-test or one-way analysis of variance (ANOVA) using the SPSS19 software program.

## Results

In this study, we aimed to determine the role of lncRNA-ADD3-AS1 in the proliferation, migration, and apoptosis of immortalized HSCs, LX-2 cells. Using adenovirus vectors, we successfully overexpressed and knockdown the lncRNA-ADD3-AS1 in LX-2 cells. The transfected cells were used to evaluate the function of lncRNA-ADD3-AS1 in cell proliferation, migration, and apoptosis.

### LncRNA-ADD3-AS1 promotes the proliferation of LX-2 cells

**Figure 1.**
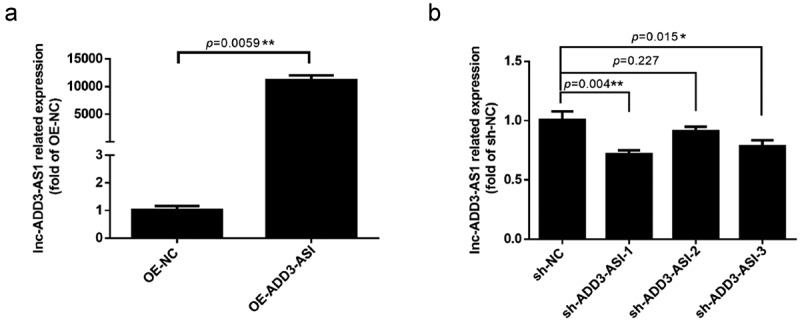
Expression levels of lnc-ADD3-AS1 in LX-2 cells transfected with designated adenovirus. (a) Fold overexpression of lnc-ADD3-AS1 in LX-2 cells, quantified by RT-qPCR. OE-ADD3-AS1, LX-2 cells infected with lnc-ADD3-AS1-expressing adenovirus; OE-NC, LX-2 cells infected with control adenovirus. The data were normalized to GAPDH. Lnc-ADD3-AS1 expression levels were further normalized to the expression level of OE-NC, defined as 1. (b) Fold change of lnc-ADD3-AS1 in LX-2 cells, quantified by RT-qPCR. Sh-NC, LX-2 cells infected with control adenovirus. Sh-ADD3-AS1, LX-2 cells infected with adenovirus that expressed shRNA targeting lnc-ADD3-AS1. The data were normalized to GAPDH. Lnc-ADD3-AS1 expression levels were further normalized to the expression level of sh-NC, defined as 1. Value in graphs represents mean ± SEM of at least three independent experiments. **P < 0.01.

LX-2 cells have been widely characterized and show the key features of hepatic stellate cytokine signaling, retinoid metabolism, and fibrogenesis, making them a suitable model for human hepatic fibrosis [27]. To investigate the impact of lncRNA-ADD3-AS1 on fibrosis in BA, appropriate adenovirus vectors were used to overexpress and knockdown lncRNA-ADD3-AS1 (OE-ADD3-AS1 and sh-ADD3-AS1, respectively) in LX-2 cells, which was confirmed using RT–qPCR. As shown in Figure 1), lncRNA-ADD3-AS1 was ~10,000-fold higher in OE-ADD3-AS1 cells than in OE-NC cells, which were transfected with the empty vector and served as a negative control. Furthermore, shRNAs targeting lncRNA-ADD3-AS1 were used to knockdown its expression. RT–qPCR results showed that among the three specific shRNAs, sh-ADD3-AS1-1 had the highest knockdown efficiency (Figure 1)). Therefore, we chose sh-ADD3-AS1-1 for subsequent functional studies.

**Figure 2.**
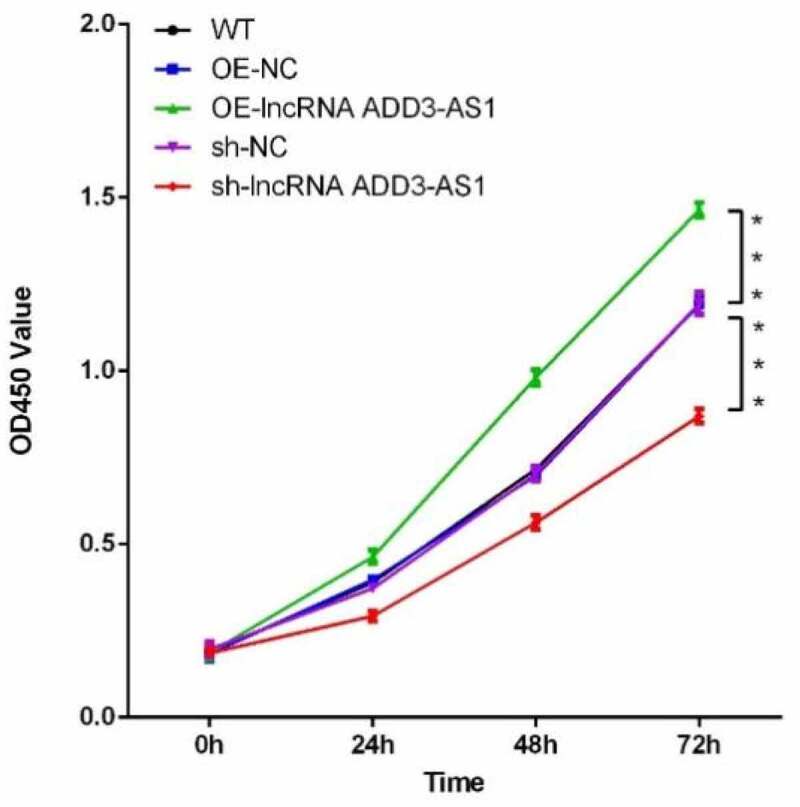
Lnc-ADD3-AS1 enhanced LX-2 cell proliferation. The transfected LX-2 cells were cultured, and total viable cells were measured with a CCK8 assay at the indicated time point. Data were presented relative to 0 h. Data are shown as mean ± SEM. ***P < 0.001.

To understand the effect of lncRNA-ADD3-AS1 on cell proliferation, we performed the CCK8 assay. We seeded LX-2 cells in 96 multi-well culture plates and transfected them with OE-ADD3-AS1 or specific sh-ADD3-AS1-1 adenovirus. Cells were then collected 0, 24, 48, and 72 h after transfection. The growth curves were examined using the CCK8 kit. As illustrated in Figure 2, the number of OE-ADD3-AS1 cells was significantly higher than the number of OE-NC cells 24 and 48 h post transfection. Conversely, the cell number remarkably decreased when lncRNA-ADD3-AS1 was partially silenced, which was consistent with the gain-of-function study. Taken together, the data showed that lncRNA-ADD3-AS1 significantly promoted the proliferation of LX-2 cells.

### LncRNA-ADD3-AS1 inhibits LX-2 cells apoptosis

**Figure 3.**
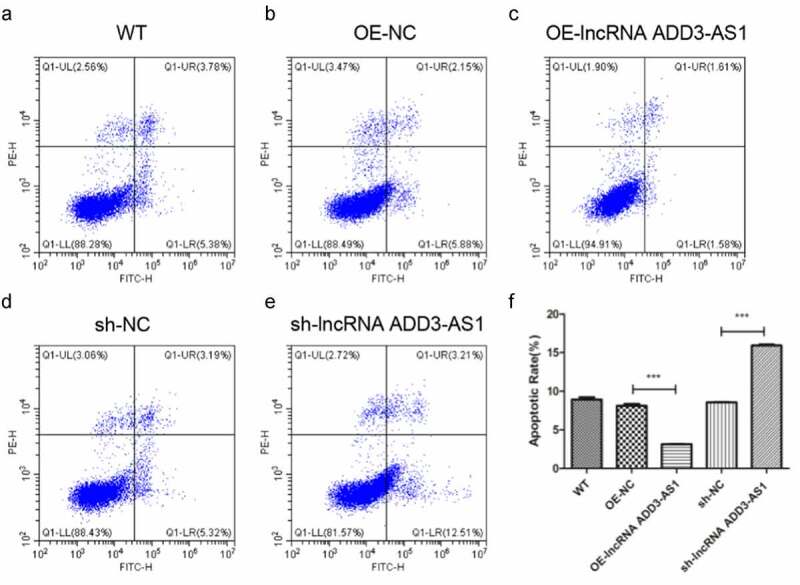
LncRNA-ADD3-AS1 inhibited the apoptosis of LX-2 cells. (a-e) The transfected LX-2 cells were obtained and flow cytometry was performed. (f) The apoptotic rate of LX-2 cells was calculated based on the data presented on panel A-E. Value in graphs represents mean ± SEM of at least three independent experiments. ***P < 0.001.

Given that lncRNA-ADD3-AS1 enhanced the proliferation of LX-2 cells, we next found out whether lncRNA-ADD3-AS1 had an impact on cell apoptosis. LX-2 cells were transfected with the indicated adenovirus, and the influence of lncRNA-ADD3-AS1 on LX-2 cell apoptosis was assessed by annexin V-FITC/PI staining and flow cytometry 24 h after transfection (Figure 3). Our data showed that lncRNA-ADD3-AS1 overexpression reduced the percentage of apoptotic cells compared to that in the control group (Figure 3), (b) and (c)), which indicated that lncRNA-ADD3-AS1 inhibited apoptosis in LX-2 cells. Consistently, apoptosis in cells treated with sh-lncRNA-ADD-AS1 adenovirus increased (Figure 3), (d) and (e)). These data confirmed that lncRNA-ADD3-AS1 attenuated LX-2 cell apoptosis.

### LncRNA-ADD3-AS1 accelerates the migration of LX-2 cells

**Figure 4.**
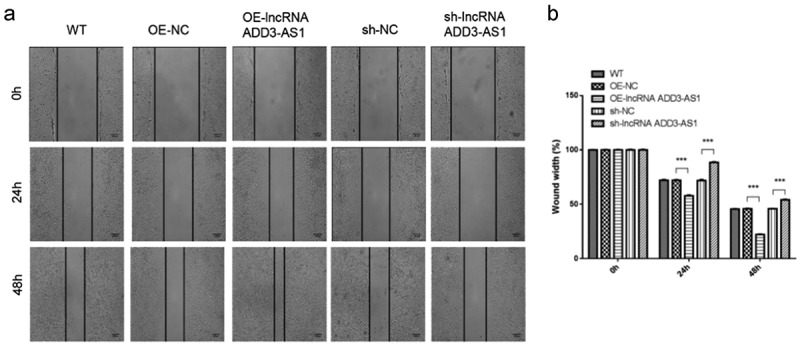
Lnc-ADD3-AS1 promoted cell migration in LX-2 cells. (a) Images of transfected cells were captured at 0, 24, and 48 h after the wound scratch. The representative images were from three independent experiments. Scale bar: 100 μm. (b) The migration potency was determined by calculating the difference in wound width. Data are shown as mean ± SEM. ***P < 0.001.

We then investigated the effect of lncRNA-ADD3-AS1 on the migration of LX-2 cells by performing wound-scratch healing and transwell migration assays. The migration ratio (%) was calculated using the wound width at designated time points. The results showed that more cells overexpressing lnc-ADD3-AS1 moved into the wound gap after 24 h and 48 h, while the number of migrating cells decreased after knocking down lnc-ADD3-AS1 (Figure 4)). As illustrated in Figure 4), the wound area was only 56% (24 h) and 21% (48 h) open in Lnc-ADD3-AS1 OE cells, whereas approximately 72% (24 h) and 45% (28 h) open gaps remained in the OE-NC group. By contrast, the open gaps in sh-ADD3-AS1-1 cells reached 89% (24 h) and 54% (48 h), which were larger than those in sh-NC cells with 70% (24 h) and 45% (48 h) open gaps.

**Figure 5.**
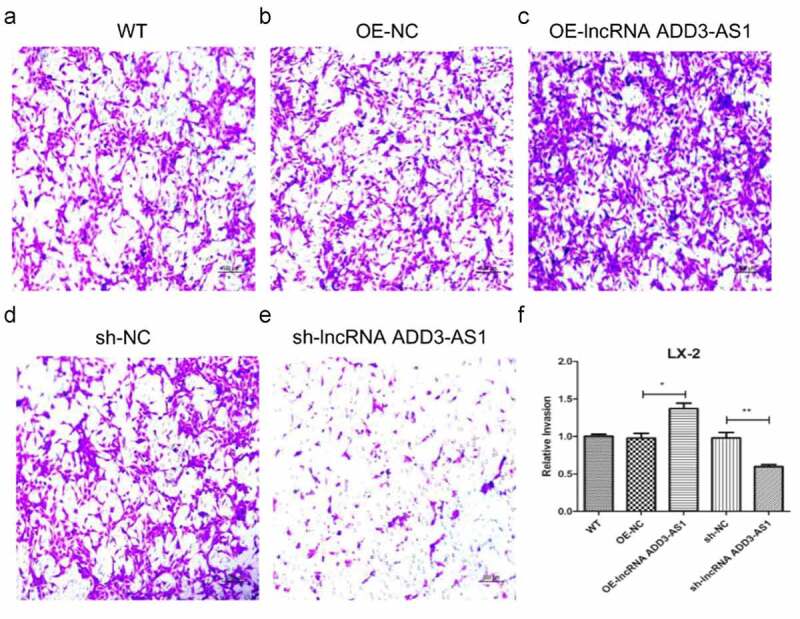
LncRNA-ADD3-AS1 promoted LX-2 cell migration. (a-e) The migration of transfected cells was tested by transwell invasion assay and migrated cells were stained purple with crystal violet. Scale bar: 100 μm. (f) Quantitative analysis of migrated cell density for panel A-E. Value in graphs represents mean ± SEM of at least three independent experiments. *P < 0.05, **P < 0.01.

For transwell migration analysis, LX-2 cells were cultured on 12-μm-pore size transwell inserts. Serum in the lower chamber served as a chemoattractant and the cells migrating through the inserts were stained and counted. As shown in Figure 5, lnc-ADD3-AS1 increased more than 1.5 times the cells that migrated toward the serum compared to that by the negative control (OE-NC) (Figure 5, c), and (f)). By contrast, the migration of sh-ADD3-AS1-1 cells was suppressed (Figure 5, e), and (f)). Our results demonstrated that lnc-ADD3-AS1 significantly accelerates LX-2 cell migration.

## Discussion

BA is the most common cause of obstructive jaundice in infants. Children with cholestasis and progressive liver fibrosis who do not receive treatment usually die before reaching two years of age [28]. Liver fibrosis in BA progresses more rapidly than any other hepatic or biliary disease in adults and children. However, the underlying pathogenic factors remain unclear. Therefore, there is an urgent need to investigate the mechanisms related to fibrosis in BA and determining the potential diagnostic or therapeutic markers. In this study, we found that lnc-ADD3-AS1 significantly promoted LX-2 cell proliferation and migration, indicating that lnc-ADD3-AS1 is correlated with liver fibrosis progression.

Liver fibrosis is the excessive accumulation of extracellular matrix (ECM) proteins occurring most types of chronic liver diseases. HSCs are the main cell type responsible for liver fibrosis because the proliferation and migration of activated HSCs amplify the fibrotic response. Here, we disrupted the expression of lnc-ADD3-AS1 in LX-2 cells and found that lnc-ADD3-AS1 overexpression significantly promoted the proliferation and migration of LX-2 cells as indicated by the CCK8 assay, transwell assay, and wound-scratch test (Figure 2,Figure 3 Figure 4, and Figure 5, respectively). Conversely, knockdown of lnc-ADD3-AS1 expression inhibited LX-2 cell proliferation and migration and promoted apoptosis in LX-2 cells. Another reason that HSCs are responsible for liver fibrosis is that they are the primary cell type, increasing the synthesis and deposition of ECM proteins in the liver. Upregulation of α-SMA and collagen-α1 proteins can be used as an indicator to determine the activation state of HSCs and liver fibrosis. We observed that lnc-ADD3-AS1 significantly upregulated the protein expression of α-SMA (Fig. S1), indicating that lnc-ADD3-AS1 could induce liver fibrosis. However, we did not observe any reduction in α-SMA expression once lnc-ADD3-AS1 was silenced, most likely because lnc-ADD3-AS1 itself had lower expression in LX-2 cells or that silencing lnc-ADD3-AS1 affected other key inducers of α-SMA.

However, the exact function of lncRNAs-ADD3-AS1 during the pathogenesis of liver fibrosis is still not known. The mechanism of action of different lncRNAs is closely linked to their interaction with RNA-binding proteins (RBPs) in the cytoplasm or nucleic acids in the nucleus. Therefore, further studies are needed to reveal lncRNAs-ADD3-AS1 localization and identify RBPs or the nucleic acids interacting with it. In the development of liver fibrosis, some lncRNAs regulate diverse cellular processes by acting as competing endogenous RNAs (ceRNAs) in the cytoplasm [10]. We hypothesize that lncRNAs-ADD3-AS1 modulates ADD3 expression as a ceRNA. LncRNAs-ADD3-AS1 has been reported as a transcript from ADD3 antisense and is associated with BA [25]. The locus on 10q25 near *XPNPEP1* and *ADD3* genes was susceptible to BA in a genome-wide SNP association study conducted in Han Chinese patients with BA and controls [20], in an independent Chinese cohort [29] and in a Thai cohort [30]. Subsequent functional studies in an animal model [22] suggested that ADD3 is the most likely BA susceptibility gene at this locus [23]. More importantly, our previous study indicated that downregulation of microRNA-145 contributes to liver fibrosis by targeting ADD3 [24]. Thus, lnc-ADD3-AS1 may contribute to liver fibrosis by regulating the expression of ADD3 via microRNA-145 sponging. A similar mechanism was found for lncRNAs RNA01134 and DHRS4-AS1. LncRNA RNA01134 accelerates hepatocellular carcinoma (HCC) progression by sponging microRNA-4784 and downregulating structure-specific recognition protein 1 [31]. The lncRNA DHRS4-AS1 ameliorates HCC by suppressing proliferation and promoting apoptosis via the miR-522-3p/SOCS5 axis [32]. Nevertheless, further studies are required to confirm the role of lnc-ADD3-AS1 in liver fibrosis by performing *in vivo* experiments and investigating the underlying mechanism. Despite these limitations, this is the first report of the role of lnc-ADD3-AS1 in fibrosis in BA.

Our results illustrate that lncRNA lnc-ADD3-AS1 contributes to liver fibrosis by promoting the proliferation and migration of HSCs. Our data suggest that lnc-ADD3-AS1 plays a role in the pathogenesis of liver fibrosis in patients with BA and may serve as a potential diagnostic marker or therapeutic target for liver fibrosis in BA.

## Conclusions

BA is a devastating liver disease, and lncRNAs have been shown to regulate cellular processes during the development of liver fibrosis in BA. Here, we showed that lnc-ADD3-AS1 significantly promotes LX-2 cell proliferation and attenuates apoptosis. More importantly, lncRNA-ADD3-AS1 significantly accelerated the migration of LX-2 cells. Our data indicates that lncRNA-ADD3-AS1 plays a role in the pathogenesis of liver fibrosis in patients with BA and may serve as a potential diagnostic marker for monitoring liver fibrosis in BA or as a therapeutic target for the disease.

## Supplementary Material

Supplemental Material

## Data Availability

The data supporting the findings of this study are available upon request from the corresponding author.

## Declaration statement

This manuscript has been read and approved by all the co-authors and representatives of the institution at which the work was carried out. All authors have agreed to submit the manuscript to the journal of *Bioengineered*.

## References

[1] Hartley JL, Davenport M, Kelly DA. Biliary atresia. Lancet. 2009;374(9702):1704–1713. 19914515 10.1016/S0140-6736(09)60946-6

[2] Muraji T, Ohtani H, Ieiri S. Unique manifestations of biliary atresia provide new immunological insight into its etiopathogenesis. Pediatr Surg Int. 2017;33(12):1249–1253. 29022092 10.1007/s00383-017-4155-7

[3] Sokol RJ, Mack C. Etiopathogenesis of biliary atresia. Semin Liver Dis. 2001;21(4):517–524. 11745039 10.1055/s-2001-19032

[4] Lampela H, Pakarinen M. [Biliary atresia]. Duodecim. 2013;129(14):1485–1493. 23961607

[5] Lakshminarayanan B, Davenport M. Biliary atresia: a comprehensive review. J Autoimmun. 2016;73:1–9. 27346637 10.1016/j.jaut.2016.06.005

[6] Nizery L, Chardot C, Sissaoui S, et al. Biliary atresia: clinical advances and perspectives. Clin Res Hepatol Gastroenterol. 2016;40(3):281–287. 26775892 10.1016/j.clinre.2015.11.010

[7] Parsons CJ, Takashima M, Rippe RA. Molecular mechanisms of hepatic fibrogenesis. J Gastroenterol Hepatol. 2007;22(1):S79–84. 17567474 10.1111/j.1440-1746.2006.04659.x

[8] Friedman SL. Mechanisms of hepatic fibrogenesis. Gastroenterology. 2008;134(6):1655–1669. 18471545 10.1053/j.gastro.2008.03.003 PMC2888539

[9] Peng H, Wan L-Y, Liang -J-J, et al. The roles of lncRNA in hepatic fibrosis. Cell Biosci. 2018;8:63. 30534359 10.1186/s13578-018-0259-6 PMC6282372

[10] He Z, Yang D, Fan X, et al. The roles and mechanisms of lncRNAs in liver fibrosis. Int J Mol Sci. 2020;21(4):1482. 10.3390/ijms21041482 PMC7073061 32098245

[11] Makhmudi A, Kalim AS, Gunadi. microRNA-21 expressions impact on liver fibrosis in biliary atresia patients. BMC Res Notes. 2019;12(1):189. 30925941 10.1186/s13104-019-4227-y PMC6441216

[12] Schmitt AM, Chang HY. Long noncoding RNAs in cancer pathways. Cancer Cell. 2016;29(4):452–463. 27070700 10.1016/j.ccell.2016.03.010 PMC4831138

[13] Yu F, Zheng J, Mao Y, et al. Long non-coding RNA APTR promotes the activation of hepatic stellate cells and the progression of liver fibrosis. Biochem Biophys Res Commun. 2015;463(4):679–685. 26043697 10.1016/j.bbrc.2015.05.124

[14] Makhmudi A, Supanji R, Putra BP, et al. The effect of APTR, Fn14 and CD133 expressions on liver fibrosis in biliary atresia patients. Pediatr Surg Int. 2020;36(1):75–79. 31549181 10.1007/s00383-019-04582-2

[15] Zheng J, Dong P, Mao Y, et al. lincRNA-p21 inhibits hepatic stellate cell activation and liver fibrogenesis via p21. FEBS J. 2015;282(24):4810–4821. 26433205 10.1111/febs.13544

[16] Tu X, Zhang Y, Zheng X, et al. TGF-beta-induced hepatocyte lincRNA-p21 contributes to liver fibrosis in mice. Sci Rep. 2017;7(1):2957. 28592847 10.1038/s41598-017-03175-0 PMC5462818

[17] Wu G, Cai J, Han Y, et al. LincRNA-p21 regulates neointima formation, vascular smooth muscle cell proliferation, apoptosis, and atherosclerosis by enhancing p53 activity. Circulation. 2014;130(17):1452–1465. 25156994 10.1161/CIRCULATIONAHA.114.011675 PMC4244705

[18] Zhang QQ, Xu MY, Qu Y, et al. TET3 mediates the activation of human hepatic stellate cells via modulating the expression of long non-coding RNA HIF1A-AS1. Int J Clin Exp Pathol. 2014;7(11):7744–7751. 25550811 PMC4270585

[19] Li XQ, Ren ZX, Li K, et al. Key anti-fibrosis associated long noncoding rnas identified in human hepatic stellate cell via transcriptome sequencing analysis. Int J Mol Sci. 2018;19(3):675. 10.3390/ijms19030675 PMC5877536 29495545

[20] Garcia-Barcelo MM, Yeung M-Y, Miao X-P, et al. Genome-wide association study identifies a susceptibility locus for biliary atresia on 10q24.2. Hum Mol Genet. 2010;19(14):2917–2925. 20460270 10.1093/hmg/ddq196 PMC2893814

[21] Cheng G, Tang CS-M, Wong EH-M, et al. Common genetic variants regulating ADD3 gene expression alter biliary atresia risk. J Hepatol. 2013;59(6):1285–1291. 23872602 10.1016/j.jhep.2013.07.021

[22] Tsai EA, Grochowski CM, Loomes KM, et al. Replication of a GWAS signal in a Caucasian population implicates ADD3 in susceptibility to biliary atresia. Hum Genet. 2014;133(2):235–243. 24104524 10.1007/s00439-013-1368-2 PMC3901047

[23] Tang V, Cofer ZC, Cui S, et al. Loss of a candidate biliary atresia susceptibility gene, add3a, causes biliary developmental defects in Zebrafish. J Pediatr Gastroenterol Nutr. 2016;63(5):524–530. 27526058 10.1097/MPG.0000000000001375 PMC5074882

[24] Ye Y, Li Z, Feng Q, et al. Downregulation of microRNA-145 may contribute to liver fibrosis in biliary atresia by targeting ADD3. PLoS One. 2017;12(9):e0180896. 28902846 10.1371/journal.pone.0180896 PMC5597134

[25] Laochareonsuk W, Chiengkriwate P, Sangkhathat S. Single nucleotide polymorphisms within Adducin 3 and Adducin 3 antisense RNA1 genes are associated with biliary atresia in Thai infants. Pediatr Surg Int. 2018;34(5):515–520. 29508064 10.1007/s00383-018-4243-3

[26] Wang X, Sun H, Zhu S. Long non-coding RNA PTAR inhibits apoptosis but promotes proliferation, invasion and migration of cervical cancer cells by binding miR-101. Bioengineered. 2021;12(1):4536–4545. 34323178 10.1080/21655979.2021.1946634 PMC8806890

[27] Aydin MM, Akcali KC. Liver fibrosis. Turk J Gastroenterol. 2018;29(1):14–21. 29391303 10.5152/tjg.2018.17330 PMC6322608

[28] Bates MD, Bucuvalas J, Alonso M, et al. Biliary atresia: pathogenesis and treatment. Semin Liver Dis. 1998;18(3):281–293. 9773428 10.1055/s-2007-1007164

[29] Zeng S, Sun P, Chen Z, et al. Association between single nucleotide polymorphisms in the ADD3 gene and susceptibility to biliary atresia. PLoS One. 2014;9(10):e107977. 25285724 10.1371/journal.pone.0107977 PMC4186752

[30] Kaewkiattiyot S, Honsawek S, Vejchapipat P, et al. Association of X-prolyl aminopeptidase 1 rs17095355 polymorphism with biliary atresia in Thai children. Hepatol Res. 2011;41(12):1249–1252. 22118303 10.1111/j.1872-034X.2011.00870.x

[31] Zheng S, Guo Y, Dai L, et al. Long intergenic noncoding RNA01134 accelerates hepatocellular carcinoma progression by sponging microRNA-4784 and downregulating structure specific recognition protein 1. Bioengineered. 2020;11(1):1016–1026. 32970959 10.1080/21655979.2020.1818508 PMC8291876

[32] Zhou Y, Li K, Zou X, et al. LncRNA DHRS4-AS1 ameliorates hepatocellular carcinoma by suppressing proliferation and promoting apoptosis via miR-522-3p/SOCS5 axis. Bioengineered. 2021;12(2):10862–10877. 34666613 10.1080/21655979.2021.1994719 PMC8809963

