# Supplementary material for: Role of long non-coding RNA-adducin 3 antisense RNA1 in liver fibrosis of biliary atresia
Source: Bioengineered. 2022 Mar 4;13(3):6222–30. doi: 10.1080/21655979.2022.2041321 (PMC8974046; doi:10.1080/21655979.2022.2041321)
Supplement: Supplemental Material [file KBIE_A_2041321_SM8376.docx]

**Supplemental Data**


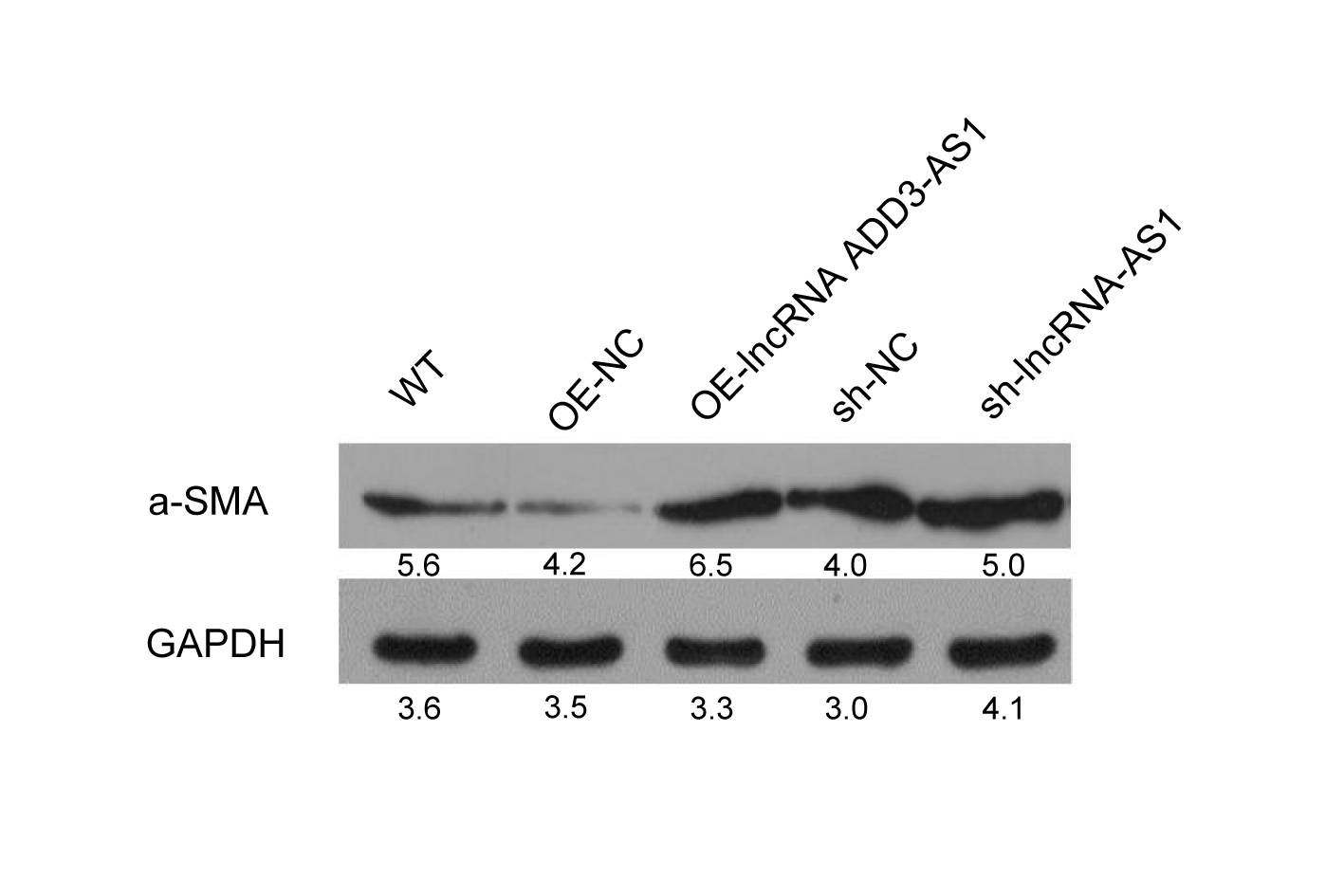


Figure S1. Western blot analysis showing that the expression of α-SMA was not significantly different in the indicated cell types. GAPDH was used as a loading control. The numbers below each blot are the relative quantification of band intensity determined by Image J.
